# Therapeutic Potential of Probiotics in Metabolic Dysfunction-Associated Steatohepatitis: A Comprehensive Review

**DOI:** 10.3390/microorganisms13081894

**Published:** 2025-08-14

**Authors:** Xueying Wang, Zhiying Wei, Qing Xiang, Lijie Tang, Weichun Xie

**Affiliations:** 1 College of Veterinary Medicine, Northeast Agricultural University, Harbin 150030, China; tycoon28644@163.com (X.W.); weizhiying1998@163.com (Z.W.); 13843891598@163.com (Q.X.); 2 Heilongjiang Key Laboratory for Animal Disease Control and Pharmaceutical Development, Northeast Agricultural University, Harbin 150030, China; 3 Key Laboratory of Dairy Science, Ministry of Education, Department of Food Science, Northeast Agricultural University, Harbin 150030, China

**Keywords:** metabolic dysfunction-associated steatohepatitis, probiotics, gut microbiome, gut–liver axis

## Abstract

Metabolic dysfunction-associated steatohepatitis (MASH) constitutes a significant and progressive liver disease, characterized by a complex pathogenesis that involves dysbiosis of the gut microbiota. While the multifaceted nature of MASH is widely recognized, its underlying mechanisms remain the subject of active investigation. Contemporary research highlights the critical role of the gut–liver axis, suggesting that disturbances in the gut microbiome may contribute to the progression of the disease. Probiotics have notably emerged as a promising therapeutic approach for MASH, with the potential to modulate the gut microbiome and mitigate symptoms. This review aims to examine the alterations in the gut microbiome associated with MASH pathogenesis, the interaction of probiotics with the gut–liver axis, and their significance in the development and management of MASH. By synthesizing current evidence on the mechanisms of action of probiotics, clinical trials, and comparative efficacy of different strains, as well as existing controversies, challenges, and future research directions, this review seeks to establish a scientific foundation for probiotic-based interventions as an innovative therapeutic strategy for MASH.

## 1. Introduction

In 2023, the terms non-alcoholic fatty liver disease (NAFLD) and non-alcoholic steatohepatitis (NASH) were updated to metabolic dysfunction-associated steatotic liver disease (MASLD) and metabolic dysfunction-associated steatohepatitis (MASH) by the medical community [1]. MASH represents a severe and progressive liver disorder, characterized by hepatocyte injury, inflammation, and fibrosis, with a significant potential to progress to advanced complications such as cirrhosis [2,3,4]. The global prevalence of MASH is increasing, largely driven by the rising incidence of obesity and metabolic syndrome, thereby imposing a substantial burden on public health systems [5,6].

The pathogenesis of MASH is multifactorial, primarily involving metabolic dysregulation and dysfunction of the gut–liver axis. Intestinal dysbiosis leads to increased intestinal permeability and the translocation of microbial components, which activate hepatic inflammatory pathways and immune responses, exacerbating tissue damage [5,7,8,9,10,11]. Despite advances in understanding the mechanisms underlying MASH, achieving a definitive diagnosis remains challenging, with liver biopsy continuing to serve as the reference standard despite its inherent limitations [12,13]. Therapeutic options are similarly limited; lifestyle modifications often suffer from poor adherence, and pharmacotherapy demonstrates variable efficacy and accessibility [2,3].

The existing diagnostic and therapeutic gaps highlight the potential of probiotics as an innovative intervention. Probiotics, defined as live microorganisms that provide health benefits to the host [14], influence the gut–liver axis by excluding pathogens competitively, enhancing the intestinal barrier, and regulating the immune system [15,16,17,18,19]. These mechanisms may address key pathological aspects of MASH, such as endotoxemia and metabolic disturbances [19].

This review offers a comprehensive analysis of gut microbiome alterations in the pathogenesis of MASH and assesses the modulation of gut–liver interactions by probiotics. We integrate evidence on mechanistic actions, clinical outcomes, and emerging innovations to establish a scientific basis for therapeutic strategies involving probiotics.

## 2. Methods

A comprehensive literature review was conducted to evaluate current strategies for the management of metabolic dysfunction-associated steatohepatitis. A systematic search was performed using PubMed, Web of Science, and Scopus employing broad key terms such as “Metabolic Dysfunction-associated Steatohepatitis”, “Probiotics”, and “Gut Microbiome”. Only studies published in English or with available English translations were ultimately included in the final analysis.

The inclusion criteria comprised articles published between 1 January 2013 and 1 August 2025, focusing specifically on metabolic dysfunction-associated steatohepatitis and probiotics. This included studies evaluating supportive care, gut–liver axis, MASH, risk factors, and gut metabolism. Studies were excluded if they were letters, editorials, commentaries, or responses to the editor, were not peer-reviewed, or were not available in English. The selection process involved a detailed screening of titles, abstracts, and references. Priority was given to studies providing high-quality, evidence-based information.

**Figure 1:**
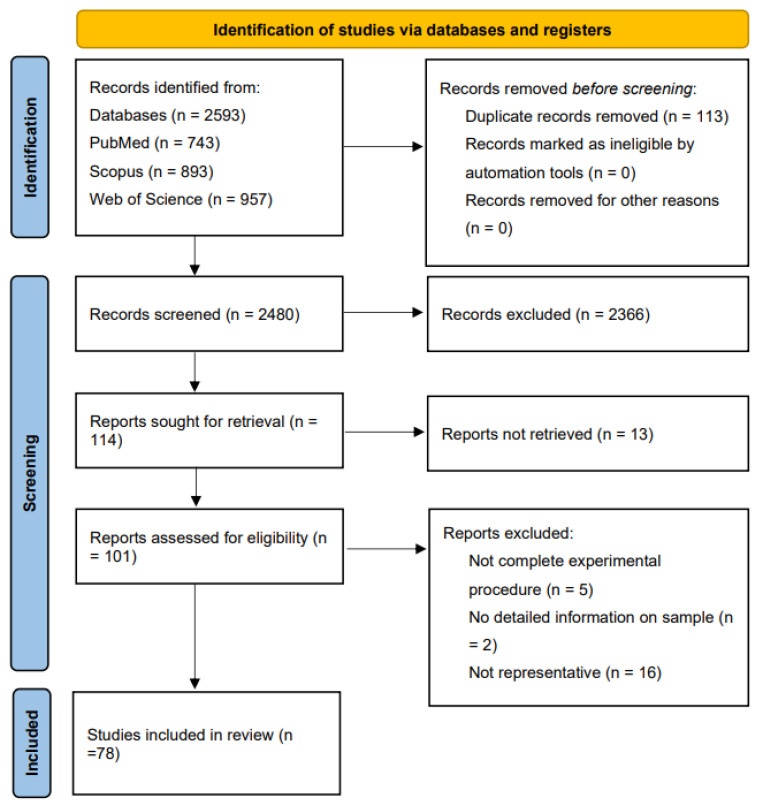
A PRISMA-style flow diagram illustrating the literature selection process for this review [20]. A total of 2593 articles were identified from PubMed, Web of Science, and Scopus. After screening and eligibility assessment, 78 studies were included in the final comprehensive literature analysis.

A total of 743 articles were screened from PubMed, of which 19 met the inclusion criteria. In total, 893 articles were screened from Scopus, with 28 ultimately included. Additionally, 957 articles were initially screened from Web of Science, of which 31 were ultimately selected for inclusion. The earliest included study was published in 2013, and the most recent study included in our paper was published in 2025 (Figure 1).

## 3. Fundamental Concepts of Probiotics and MASH

### 3.1. Definition, Classification, and Mechanisms of Probiotics

Probiotics are live microorganisms that, when administered in sufficient quantities, provide health benefits to the host [14]. They exert both direct and indirect effects on various physiological systems, with a particular emphasis on the gastrointestinal system [21]. Probiotics can be categorized into various genera, with *Lactobacillus*, *Bifidobacterium*, and *Enterococcus* being among the most extensively studied. For instance, *Bifidobacterium* strains isolated from patients with inflammatory bowel disease (IBD) were characterized, revealing that *B. longum* exhibits significant probiotic potential due to its ability to synthesize exopolysaccharides, its antibacterial activity, and its anti-inflammatory properties [22].

Probiotics can be sourced from traditional foods like kimchi and pearl millet porridge, where the strains KAC1 and PAC1 were found [23]. These Enterobacteriaceae strains can withstand gastric juice and inhibit pathogenic bacteria [23]. Furthermore, the taxonomic classification of certain probiotics has undergone re-evaluation. *Enterococcus* faecium SF68 was reclassified as *Enterococcus* lactis based on phylogenomic and average nucleotide identity (ANI) analyses, and it was identified as having the potential to produce bacteriocin and inhibit the growth of specific pathogens [24].

Probiotics play a crucial role in regulating the gut microbiome through various mechanisms. They compete with harmful bacteria for nutrients and adhesion sites, reducing pathogen colonization [15]. Probiotics produce antimicrobial substances like bacteriocins and organic acids to inhibit pathogen growth [16]. They also interact with immune cells to influence cytokine production, as shown in a study on irritable bowel syndrome (IBS) where probiotics alleviated symptoms and inflammation [17]. Additionally, probiotics strengthen gut barrier integrity by increasing tight junction protein expression, preventing bacteria and toxins from entering the bloodstream [15].

### 3.2. Pathological Features and Clinical Progression of MASH

**Figure 2:**
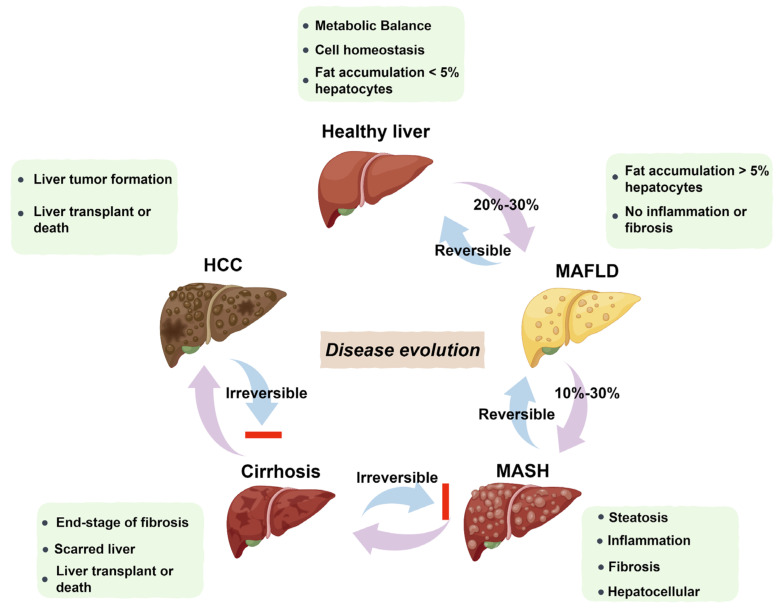
Pathogenesis and progression of MAFLD and MASH. MAFLD begins with fat accumulation and can progress to MASH with additional inflammation. NASH involves fibrosis and liver cell death. Severe fibrosis leads to cirrhosis, causing permanent liver damage. In some cases, cirrhosis advances to HCC. While MAFLD and MASH can be reversed in a healthy liver, cirrhosis and HCC are irreversible.

**Figure 3:**
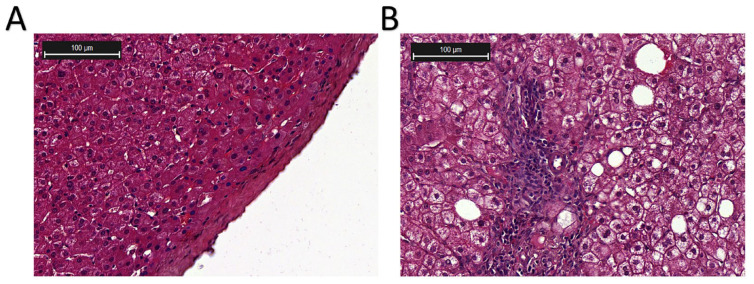
Histological examination of the liver biopsies (image cited from [28]). (**A**) Liver biopsy from a healthy donor. (**B**) Liver biopsy from a MASH patient. Hematoxylin and eosin (H & E) staining.

More than a quarter of adults worldwide are affected by MASLD, which can develop into MASH, increasing the likelihood of liver fibrosis, cirrhosis, hepatocellular carcinoma (HCC), and cardiovascular complications [25]. The pathogenesis and progression of MAFLD and MASH are shown in Figure 2 [26,27]. Histologically, MASH is marked by hepatocyte ballooning, macrophage polarization, ductular reaction, and activation of hepatic stellate cells [4]. At the cellular level, MASH is associated with pronounced cellular plasticity, with hepatocytes, macrophages, cholangiocytes, and hepatic stellate cells (HSCs) undergoing significant intracellular alterations in response to extracellular stimuli [4]. While liver biopsy remains the diagnostic gold standard, non-invasive diagnostic methods are under development [12,13]. Histological analysis of liver biopsies from healthy donors showed no inflammation or structural changes, with prominent central and portal veins and minimal intracellular cholestasis (Figure 3A) [28]. In contrast, liver biopsies from MASH patients (Figure 3B) revealed increased fatty deposits, more lymphocytes, and hepatocyte dystrophy [28].

In terms of treatment, lifestyle modifications are currently the primary approach; however, there is a significant need for effective pharmacotherapies. The study of herbal extracts and natural products as treatments for MASH is advancing due to their diverse pharmacological effects and minimal side effects. The induction of autophagy through the use of herbal extracts and natural products represents a promising therapeutic mechanism, as it addresses hepatic steatosis, inflammation, oxidative stress, and apoptosis, which are all pivotal factors in the progression of MASH [29]. Herbal extracts and natural products also target macrophage-mediated inflammation, crucial in MASH, by modulating macrophage activity to reduce liver inflammation and fibrosis [30,31]. Additionally, human placental extract (HPE) shows promise in treating MASH by reducing hepatic fibrosis and early cirrhosis through its immunotropic, anti-inflammatory, and antioxidant effects, potentially preventing MASH progression [32]. L-carnitine may improve liver health and lower hepatocellular carcinoma risk in MASH by boosting mitochondrial function and reducing inflammation [33]. Ongoing research and clinical trials are crucial to understanding their mechanisms and optimizing their clinical use, potentially offering effective, accessible treatments for MASH [34,35]. In a phase II clinical trial, Heptex^®^ capsules, containing extracts of Phyllanthus niruri and Silybum marianum, were tested in patients with MASH risk factors. The findings demonstrated a notable reduction in alanine aminotransferase (ALT) levels in both the low-dose and high-dose groups, and a decrease in aspartate aminotransferase (AST) levels in the high-dose group, suggesting potential hepatoprotective effects; however, further evaluation is warranted [2]. Resmetirom, a thyroid hormone receptor agonist, has received FDA approval for the treatment of MASH. In clinical trials, it achieved MASH resolution in 24.2% and 25.9% of patients at doses of 80 mg and 100 mg, respectively, compared to 14.2% in the placebo group, and also demonstrated improvements in liver fibrosis [3].

### 3.3. Evidence-Based Evolution of Probiotics in Liver Disease

The use of probiotics in liver disease treatment has a progressively evolving history. Recently, with the recognition of the gut–liver axis, the role of probiotics in liver diseases has received increased attention [36,37]. In the context of treating alcoholic liver disorders, probiotics such as *Lactobacillus* and *Bifidobacteria* have been investigated for their potential to alleviate alcohol-related liver diseases [38]. These probiotics exert their effects through mechanisms such as modulating the gut microbiome, enhancing intestinal barrier function and immune response, reducing endotoxin levels, and preventing bacterial translocation [38]. In the case of alcoholic liver disease, a meta-analysis of nine randomized controlled trials (RCTs) indicated that probiotic preparations could significantly improve liver function by reducing levels of ALT, AST, and γ-glutamyl transpeptidase, while increasing serum albumin levels [39]. In the context of cirrhotic liver disease, a systematic review and meta-analysis of 22 RCTs demonstrated that probiotics significantly reduced levels of Gamma-glutamyl transferase (GGT), AST, serum ammonia, and endotoxins, indicating their potential as an adjunctive therapy [40].

Similarly, the role of probiotics in MASLD and MASH has been investigated. Although the precise mechanisms remain under investigation, probiotics are believed to enhance epithelial barrier function, inhibit bacterial translocation, and strengthen the immune system, potentially benefiting patients with these liver conditions [37]. Dysbiosis of the gut microbiota has been observed in patients with MASLD/MASH, and probiotics have been hypothesized to restore microbial balance. For example, a study on MASLD highlighted the critical role of the gut microbiota in its pathogenesis [41]. These findings indicate that probiotics have shown promise in the management of liver diseases, but more research is needed to optimize their use.

## 4. Epidemiology and Pathophysiology of MASH

### 4.1. Global Burden, Risk Factors, and Prevalence Trends

The global prevalence of MASH is increasing, thereby imposing a substantial public health burden. An analysis of data from the Global Burden of Disease Study 2021 revealed a rise in the incidence of new cases, mortality, and disability-adjusted life years (DALYs) attributable to MASH-related liver cancer from 1990 to 2021 [6]. In 2021, there were 42,291 new cases, 40,925 deaths, and 995,475 DALYs associated with MASH-related liver cancer worldwide [6].

Several risk factors are linked to the development of MASH. Obesity, particularly abdominal obesity, is a primary risk factor, while diabetes significantly elevates the risk. Additional risk factors include hypertension, dyslipidemia, and certain genetic predispositions. The impact of risk factors on MASH is shown in Table 1. Furthermore, these risk factors lead to changes in the gut microbiota (Table 2), and the pathogenesis of MASH is associated with dysbiosis of the gut microbiota. Modulating metabolism via the gut microbiota constitutes a promising approach for the prevention and treatment of MASH, and its associated risk factors, including obesity and diabetes.

Within the Iranian adult population of the Pars Cohort Study, being overweight or obese was independently correlated with an increased risk of MASH, with an odds ratio (OR) of 2.192, and the prevalence of MASH among adults aged 40 to 75 years was found to be 6.9% [42]. The study identified male gender, younger age, a history of heart disease, diabetes, hypertension, higher socioeconomic status, and obesity as risk factors for MASH within this population [42]. In a population-based study, the OR for developing MASH among individuals with diabetes was 3.55, after adjusting for confounding variables [43]. Furthermore, in patients with *Helicobacter pylori* infection, the OR for developing MASH was 2.51, suggesting an association between the infection and MASH [43]. Another study, which examined 968 patients with type 2 diabetes mellitus, revealed that hyperferritinemia was significantly more prevalent in the MASLD group compared to the non-MASLD group (83.3% vs. 56.3%) [44]. Furthermore, hyperferritinemia was associated with advanced liver fibrosis in MASLD patients, particularly among males [44]. Additionally, in lean adults in the United States, the age-adjusted prevalence of high-risk MASH was 1.29%, with older age and metabolic comorbidities such as hypertension, diabetes, and dyslipidemia being associated with MASH and its complications [45].

**Table 1:** Impact of risk factors on MASH.

Risk Factors	Pathological Mechanism	Key Effects	References
Obesity	Adipose tissue hypoxia, inflammatory cytokine release, free fatty acids	Hepatic lipid accumulation, Kupffer cell activation, inflammatory reaction	[46]
Diabetes	Insulin resistance, impaired hepatic insulin clearance	Chronic hyperinsulinemia, hepatic stellate cell activation	[47]
Hypertension	Renin–angiotensin system activation, hepatic vasoconstriction/hypoxia	Hepatocyte injury, fibrogenic pathway activation	[48,49]
Dyslipidemia	Cholesterol-laden lipid droplets, lysosomal membrane permeabilization	Inflammasome activation	[50]
Genetic predispositions	Lipid droplet homeostasis disruption	Lipid droplet rupture, lipotoxic mediator release, mitochondrial dysfunction	[51,52,53]

**Table 2:** Association between risk factors and gut microbiome alterations.

Risk Factors	Gut Microbiome Alterations	Key Metabolic/Immune Consequences	References
Obesity	α-diversity ↓, *Faecalibacterium prausnitzii* ↓, *Butyrate-producing bacteria* ↓, *Escherichia coli* ↑	Hepatic glycolipid deposition ↑, insulin resistance ↑, SCFAs ↓, D-lactate ↑	[54,55]
Diabetes	*Bacteroides* ↓, *Faecalibacterium prausnitzii* ↓	GLP-1 secretion ↓, hepatic gluconeogenesis ↑, RORDEPs ↓	[56,57,58]
Hypertension	*Roseburia* ↓, *Streptococcus salivarius* ↑, *Eggerthella lenta* ↑, *Erysipelatoclostridium ramosum* ↑	Dysregulated blood pressure control, vascular inflammation ↑, fecal acetate/propionate ratio ↑	[59,60]
Dyslipidemia	*Bifidobacterium* ↓, Shannon ↓, Ruminococcus gnavus ↑	β-oxidation ↓, NLRP3 inflammasome activation, TMAO ↑	[61,62]
Genetic predispositions	α-diversity ↓, fecal acetate/propionate ratio, *Saccharimonadaceae* ↑	LPS ↑→inflammation, secondary bile acid ↑→oxidative stress	[55,63]

↑, upregulate; ↓, downregulate; →, entailment relations, results.

### 4.2. Metabolic Dysregulation and Inflammatory Signaling

The pathophysiology of MASH is intricate, involving a multitude of factors. It is primarily characterized by hepatic steatosis, inflammation, hepatocyte injury, and fibrosis [5]. Insulin resistance is recognized as a significant contributor to hepatic steatosis [7]. A pivotal role is played by lipotoxicity, wherein an excess of free fatty acids within the liver induces oxidative stress, mitochondrial dysfunction, and endoplasmic reticulum stress, culminating in hepatocyte damage and inflammation [7]. The accumulation of lipids in hepatocytes, a hallmark of lipotoxicity, precipitates oxidative stress, endoplasmic reticulum stress, and the activation of inflammatory pathways [64].

Inflammatory pathways are also of paramount importance. The activation of toll-like receptor 4 (TLR4) signaling by lipopolysaccharides (LPSs) derived from the gut microbiota can initiate an inflammatory cascade within the liver, leading to the production of pro-inflammatory cytokines such as tumor necrosis factor-alpha (TNF-α) and interleukin-1β (IL-1β) [8]. Furthermore, the activation of the NLRP3 inflammasome has been implicated in MASH, facilitating the release of IL-1β and IL-18, which further intensify inflammation [9].

Similarly, research examining the FOXM1/CMA/ER stress axis in MASH revealed significantly reduced hepatic chaperone-mediated autophagy (CMA) activity in both MASH mice and patients [65]. The deficiency of CMA in hepatocytes was shown to promote fibrosis and hepatic inflammation by inducing cholesterol accumulation and endoplasmic reticulum (ER) stress [65]. Additionally, another study found that the inhibition of ZFP281/ZNF281-RIPK1/RIPK3/MLKL signaling in hepatocytes through pterostilbene alleviated hepatic lipometabolic disorder and inflammation in MASH [66]. In this context, elevated levels of ZNF281 were associated with the upregulation of pro-inflammatory signaling and the disruption of mitochondrial fatty acid β-oxidation, thereby facilitating lipid accumulation and pro-inflammatory cell death [66].

### 4.3. Gut–Liver Axis Crosstalk in MASH Development

The gut–liver axis is a pivotal concept in the comprehension of MASH, encompassing a complex bidirectional communication between the gut microbiota and the liver. The gut microbiome constitutes a complex ecosystem of trillions of microorganisms that are integral to maintaining host health. In the context of MASH, dysbiosis of the gut microbiota has been implicated in the disease’s pathogenesis. Research indicates that alterations in the composition and diversity of the gut microbiome can result in metabolic disturbances, increased intestinal permeability (allowing bacterial products such as LPS to reach the liver), and immune system activation, all of which contribute to the development and progression of MASH [10,11].

For example, a study involving mice on a high-fat diet demonstrated that the administration of Alistipes indistinctus inhibited the progression from MASLD to MASH by enhancing the gut barrier and increasing the abundance of *Lactobacillus* species [67]. This finding suggests that specific gut bacteria may exert a protective effect against MASH. A comparative study of the gut microbiota in patients with metabolic dysfunction-associated steatotic liver (MASL) and MASH revealed a notable reduction in intestinal flora diversity during the progression of MASLD. This was accompanied by shifts in the abundance of certain phyla, including Bacteroidetes, Firmicutes, and Fusobacteria, and genera such as *Prevotella*, *Megamonas*, and *Fusobacterium* [68].

Furthermore, metabolites derived from gut microbiota, such as short-chain fatty acids and bile acids, have the capacity to modulate hepatic metabolism and inflammation. These metabolites can either exacerbate or mitigate MASH depending on their specific composition and concentration [69]. The gut microbiota also plays a crucial role in bile acid metabolism, with changes in bile acid composition potentially contributing to the pathogenesis of MASH.

For instance, in a murine model of MASH induced by a high-fat diet, the administration of ginger essential oil was found to inhibit MASH progression by suppressing the NLRP3 inflammasome and modulating the gut microbiota-LPS-TLR4 pathway, which led to a reduction in plasma ALT levels and hepatic pro-inflammatory cytokine levels [9]. Similarly, a study investigating the effects of tomato juice (TJ) on MASH demonstrated that TJ supplementation ameliorated gut microbiota dysbiosis caused by a methionine–choline-deficient diet in mice. This supplementation increased the abundance of bacteria producing short-chain fatty acids and succinic acid, and it enhanced the expression of proteins associated with the intestinal barrier [70]. This resulted in the inhibition of MASH progression through the reduction in serum lipid concentrations, the alleviation of endotoxin levels, and the suppression of the activation of the endotoxin-TLR4-NF-κB pathway [70]. A study investigating the role of gut microbiota and bile acid metabolism in the mechanism of ginsenoside Re against MASLD demonstrated that ginsenoside Re modulated microbial genera associated with bile salt hydrolase, thereby altering bile acid synthesis pathways and inhibiting MASLD progression, which may also be pertinent to MASH [71].

### 4.4. Dietary Interventions for MASH: Impact on Gut Microbiota and Liver Health

Research on diet and probiotic regulation examines how dietary choices affect gut microbiota and overall health. A study on golden hamsters with high-fat diet-induced MASLD found that probiotic yogurt improved lipid metabolism and gut microbiota [72]. It reversed liver steatosis and oxidative stress, reduced the Firmicutes/Bacteroidetes ratio, and increased short-chain fatty acid-producing bacteria [72]. This indicates that probiotics may be a promising nutritional approach for managing MASLD.

The therapeutic potential of dietary interventions is further substantiated by research demonstrating the benefits of specific dietary components. For example, a ketogenic diet based on vegetable oils has been shown to ameliorate liver inflammation and fibrosis in experimental models of MASH, indicating that dietary fats can influence liver health through their impact on gut microbiota and metabolic pathways [73]. Furthermore, the impact of diet quality and physical activity on MASLD and MASH has been underscored, with high-quality diets (evaluated by factors such as the intake of protein foods, whole grains, dairy, fruits, vegetables, saturated fat, and added sugars in accordance with the Dietary Guidelines for Americans) and increased physical activity being associated with lower risks of these conditions [74].

Dietary interventions, including fasting and caloric restriction, have the potential to modulate gut microbiota and ameliorate health conditions such as obesity, diabetes, and inflammatory bowel disease [75]. While weight loss achieved through dietary restriction leads to significant improvements in MASH markers, such as liver fat content and inflammation, the alterations in gut microbiota are not sustained, underscoring the intricate interplay between diet, microbiota, and liver health [76]. Studies show that these dietary changes, along with probiotics, enhance microbial diversity and beneficial bacteria, especially in high-fat diet populations [77]. While dietary interventions alone have a significant impact, combining them with probiotics offers a synergistic effect, suggesting a complementary approach to managing gut health and metabolic issues [77]. Additionally, the role of *Helicobacter pylori* infection in MASH introduces another layer of complexity to the gut–liver axis. Studies have demonstrated a significant association between *Helicobacter pylori* infection and an increased risk of developing MASH, suggesting that eradicating this bacterium could serve as a potential therapeutic strategy [78,79]. This underscores the importance of considering both microbial and dietary factors in the management of MASH.

## 5. Therapeutic Potential of Probiotics in MASH

### 5.1. Mechanisms of Action of Probiotics in Liver Health

#### 5.1.1. Probiotics Against High-Fat Diet Effects: Mechanisms Involving Gut Microbiota Modulation and Metabolite Production

**Figure 4:**
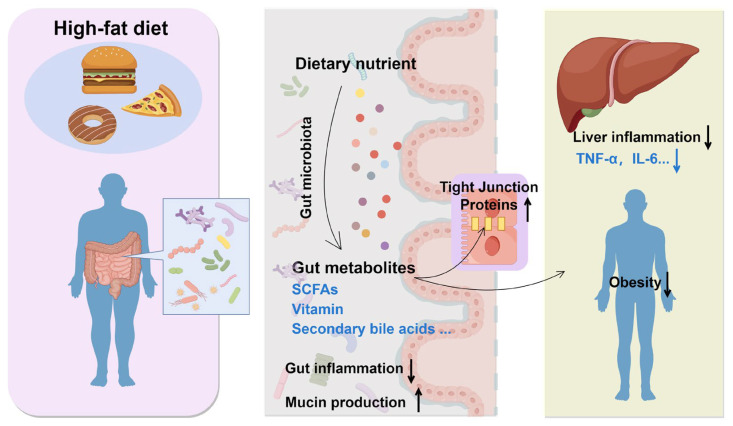
Effect of probiotics on high-fat diet. ↑, upregulate; ↓, downregulate.

Probiotics are gaining recognition for their potential role in modulating the effects of high-fat diets, particularly in relation to obesity and metabolic disorders. The effect of probiotics on a high-fat diet is presented in Figure 4. The gut microbiota plays a crucial role in maintaining energy balance, lipid metabolism, and inflammation, all of which are pivotal in the development of obesity. It also influences liver health through the production of microbial enzymes that impact the metabolism of steroids, fatty acids, and other compounds, thereby contributing to liver inflammation and fibrosis [80]. Emerging research suggests that probiotics may mitigate the adverse effects of high-fat diets by altering the gut microbiota and enhancing metabolic health. For instance, multi-strain probiotics have demonstrated efficacy in reducing weight gain and improving body fat composition in mice subjected to high-carbohydrate diets associated with obesity [81]. Furthermore, probiotics have the capacity to restore beneficial gut bacteria, such as *Bifidobacterium* and *Lactobacillus*, which are linked to lower obesity rates [82]. Alterations in the gut microbiota are correlated with enhanced metabolic parameters, including decreased triglyceride levels and improved glucose tolerance, thereby underscoring the potential of probiotics as a dietary intervention for obesity and metabolic disorders [83,84].

Beyond modifying the gut microbiota composition, probiotics also affect the production of metabolites essential for metabolic health. For instance, the intake of dietary fibers, which are frequently fermented by gut bacteria into SCFAs, has been demonstrated to enhance metabolic homeostasis [85]. Probiotics can augment the production of SCFAs, such as butyrate, which possess anti-inflammatory properties and contribute to the maintenance of gut barrier integrity [86]. This is particularly significant in the context of high-fat diets, which are known to compromise gut barrier function and promote inflammation [87].

#### 5.1.2. Strain-Specific Probiotics Modulate Microbiota for Liver Health

Probiotics confer beneficial effects on liver health through various mechanisms, with a primary mechanism being the modulation of the gut microbiota. By altering the gut microbiota composition, probiotics can decrease the prevalence of pathogenic bacteria while enhancing the presence of beneficial ones. For instance, in a study involving mice with MASH, the administration of *Faecalibacterium prausnitzii*, a promising next-generation probiotic candidate, was shown to mitigate MASH symptoms. Analyses using 16S rRNA sequencing revealed a significantly reduced relative abundance of *F. prausnitzii* in patients with MASH compared to healthy controls. In the MASH mouse model, supplementation with *F. prausnitzii* improved glucose homeostasis, prevented hepatic lipid accumulation, reduced liver damage and fibrosis, restored compromised gut barrier functions, and alleviated hepatic steatosis and liver inflammation [88]. Similarly, in a study on alcoholic liver disease, probiotics were found to modulate the gut microbiota by increasing the abundance of *Bifidobacteria* and decreasing the presence of *Escherichia coli* in patients [39]. Furthermore, research on MASH demonstrated that both single-strain and mixed-strain probiotics could effectively alter the gut microbiota composition. The relative abundance of *Lactobacillus* was observed to increase in the groups treated with probiotics, whereas the relative abundance of *Akkermansia* decreased. These alterations in the gut microbiota were correlated with the suppression of inflammatory responses in the liver, suggesting an interaction between probiotics, gut microbiota, and the host immune response [89].

It is important to note that different probiotic strains exert distinct effects on the modulation of the gut microbiome. For instance, a study comparing three strains of *Bifidobacterium* adolescentis identified *B. adolescentis* FJSSZ23M10 as the most effective in mitigating obesity induced by a high-fat diet [90]. This particular strain significantly reduced weight gain, improved abnormal serum biochemical markers, decreased lipid accumulation in adipocytes, and enhanced energy expenditure [90]. Genomic analysis revealed that *B. adolescentis* FJSSZ23M10 possessed the highest abundance of unassigned genes and carbohydrate-active enzymes (CAZymes) compared to the other strains, which may contribute to its superior functional potential in modulating the gut microbiota and metabolism [90].

In a separate study, a multi-strain probiotic consortium (MSPC) derived from dairy sources was assessed for its capacity to influence obesity-associated gut microbiota in both lean and obese Pakistani participants [91]. The impact of an MSPC was found to be individualized, resulting in a reduction in bacterial counts in certain lean samples while increasing them in some obese samples. Notably, an MSPC significantly enhanced α-diversity in several instances and altered the composition of the gut microbiota, with beneficial bacteria such as *Bifidobacterium* and *Lactobacillus* showing a marked increase in the obese cohort [91]. These results underscore the importance of selecting appropriate probiotic strains for effective modulation of the gut microbiome in MASH.

#### 5.1.3. Probiotic-Host Immune System Interactions

Probiotics engage with the host immune response through various mechanisms, one of which involves the regulation of host microRNAs (miRNAs). Probiotics can influence the expression of host miRNAs, which are non-coding RNA molecules that govern essential biological processes, including cell growth, differentiation, and apoptosis. By interacting with messenger RNAs (mRNAs), miRNAs can either facilitate their degradation or inhibit their translation, thereby regulating gene expression post-transcriptionally and modulating the immune system [92].

Probiotics have the potential to modulate the host autophagy process, a lysosome-dependent protein degradation mechanism essential for maintaining host homeostasis. Disruptions in autophagy can result in a variety of health issues. Probiotics contribute to the preservation of host homeostasis by stimulating the immune system and influencing numerous physiological and pathological responses through the autophagy pathway [93].

Moreover, probiotics have been demonstrated to modulate immune responses, which are frequently dysregulated in the context of obesity. For example, probiotics have the capacity to restore the function of invariant natural killer T (iNKT) cells within adipose tissue, which are integral to the prevention of diet-induced obesity and its associated disorders [94]. Through the modulation of the immune system, probiotics can attenuate adipose tissue inflammation and enhance insulin sensitivity, thereby alleviating the detrimental effects associated with high-fat diets [95]. In research investigating the impact of probiotics on MASLD, it was observed that probiotics downregulated the expression of pro-inflammatory cytokines, including TNF-α and IL-6, while upregulating anti-inflammatory cytokines such as IL-10 [96]. Furthermore, probiotics enhance intestinal barrier function, thereby reducing intestinal permeability and preventing the translocation of bacterial endotoxins into the liver. In a mouse model of MASH, rifaximin was found to mitigate MASH by restoring gut, reversing intestinal barrier dysfunction, and modulating the expression of peroxisome proliferator-activated receptor (PPAR)α and PPARγ in the liver [97].

Probiotics also have the potential to influence critical signaling pathways, such as the PI3K/Akt/mTOR pathway. A study examining the effects of probiotics on human dermal fibroblasts revealed that a probiotic mixture, BioK, promoted fibroblast migration, which was associated with the upregulation of genes involved in the PI3K signaling pathways, including Paxillin, PI3K, PKC, and ITG-β1 [98].

The NF-κB signaling pathway represents a critical target for probiotic intervention. In the context of mastitis, a prevalent inflammatory disorder of the mammary gland, the activation of toll-like receptors (TLRs), particularly TLR2 and TLR4, triggers the production of pro-inflammatory cytokines via the NF-κB signaling pathway. Bioactive compounds and probiotics have been recognized as promising therapeutic agents for the prevention and treatment of mastitis by modulating the TLR2/TLR4/NF-κB signaling pathway [99].

**Figure 5:**
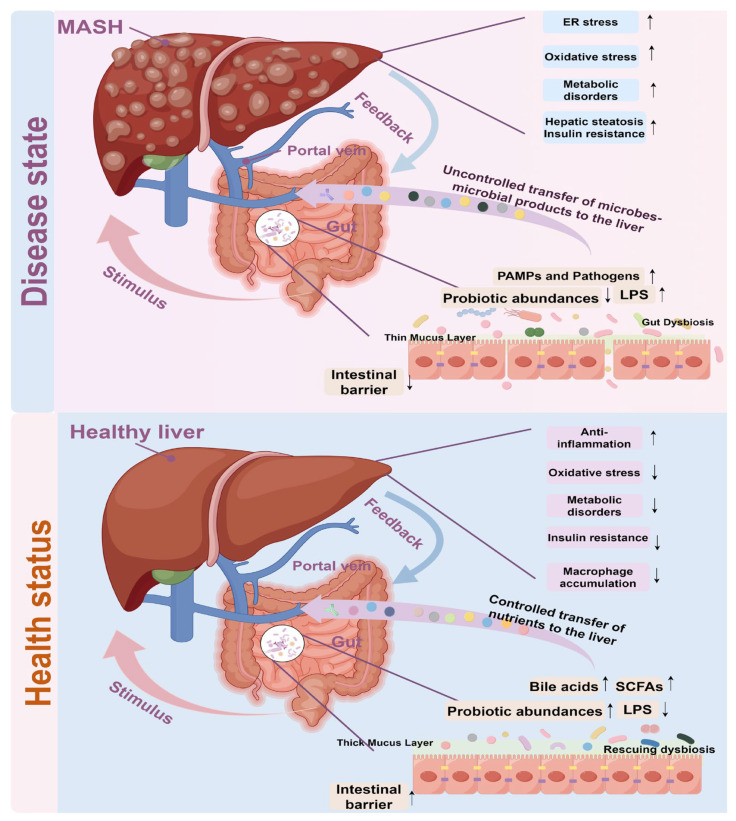
The primary mechanism of action of probiotics in liver health. ↑, upregulate; ↓, downregulate.

Similarly, in the case of MASH, probiotics may influence these pathways to attenuate inflammation and enhance liver function. For instance, a study investigating tris (2-chloroethyl) phosphate (TCEP)-induced metabolic disorder in mice demonstrated that dietary complex probiotics conferred protection against hepatic steatosis through the FXR-mediated signaling pathway. The probiotics inhibited TCEP-induced ileal FXR signaling and upregulated the hepatic FXR/SHP pathway, which was suppressed by TCEP, thereby promoting PPARα-mediated lipid oxidation and inhibiting SREBP1c/PPARγ-mediated lipid synthesis [100]. Furthermore, research has assessed the potential therapeutic effects of the genetically engineered probiotic Zbiotics (ZB183) on MASH. The oral administration of Zbiotics over a four-week period resulted in the downregulation of the cGAS-STING-related network (including MAPK3, EDN1, TNF, miR-6888-5p miRNA, and lncRNA RABGAP1L-DT-206) in models of MASH. Furthermore, Zbiotics significantly alleviated hepatic inflammation and steatosis, as demonstrated by an improvement in the MASLD Activity Score (NAS) and a reduction in hepatic TNF-α levels [101]. The primary mechanism of action of probiotics in liver health is shown in Figure 5.

### 5.2. Clinical Trial Evidence: Efficacy and Controversies

Recent clinical trials have investigated the potential of probiotics in the treatment of MASH. In the PROBILIVER randomized clinical trial, 44 adult outpatients with biopsy-confirmed MASH were assigned to receive either a probiotic mixture (comprising *Lactobacillus acidophilus*, *Lactobacillus rhamnosus*, *Lactobacillus paracasei*, and *Bifidobacterium lactis*) or a placebo for a duration of 24 weeks [102]. Although no significant differences were observed between the probiotic and placebo groups concerning metabolic syndrome, waist circumference, BMI scores, or liver enzyme levels, the probiotic group exhibited a larger effect size in the reduction in cytokeratin 18.

A double-blind, placebo-controlled trial was conducted to assess the impact of probiotic supplementation on hepatic fibrosis, inflammatory and metabolic markers, and gut microbiota in patients with MASH. A cohort of 48 MASH patients was randomly assigned to receive either probiotics (comprising *Lactobacillus acidophilus* at 1 × 10^9^ CFU and *Bifidobacterium lactis* at 1 × 10^9^ CFU) or a placebo daily for a duration of six months. The primary outcome, the APRI score, demonstrated a reduction over time in the probiotic group. However, no statistically significant differences were observed between the groups in terms of liver fibrosis, steatosis, or inflammatory activity. Additionally, the probiotic treatment did not result in substantial alterations in gut microbiota composition [103].

A proof-of-concept study was conducted in which patients with histologically confirmed MASH were randomized to receive either probiotics or standard care for a duration of six months. The group receiving probiotics demonstrated a significant reduction in intrahepatic triglyceride (IHTG) content, decreasing from 22.6 ± 8.2% to 14.9 ± 7.0%, whereas the IHTG levels remained unchanged in the group receiving usual care. Additionally, the probiotic group exhibited a more pronounced reduction in serum AST levels [104]. A meta-analysis encompassing 34 studies with a total of 12,682 participants demonstrated that supplementation with probiotics, prebiotics, and synbiotics significantly ameliorated liver injury, as evidenced by reductions in hepatic fibrosis, AST levels, ALT levels, and alkaline phosphatase levels. Furthermore, these interventions improved lipid profiles, as indicated by a reduction in triglycerides, and modulated inflammatory markers, including high-density lipoprotein and tumor necrosis factor alpha, in patients with MASLD, with potential implications for MASH [105].

Additionally, a systematic review and network meta-analysis of 37 RCTs involving 1921 MASLD patients revealed that probiotics and synbiotics were significantly associated with reductions in ALT levels and liver stiffness measurements via elastography [106]. Probiotic supplementation was also linked to significant decreases in BMI and the homeostasis model assessment of insulin resistance (HOMA-IR). Further RCTs are necessary to ascertain the efficacy of fecal microbiota transplantation and antibiotic interventions in the management of MASLD, and to investigate the incidence of adverse events associated with microbiota-targeted therapies.

Notably, the existing literature does not uniformly support positive outcomes. For instance, a double-blind, placebo-controlled, single-center study involving 46 patients with biopsy-confirmed MASH demonstrated that a 24-week regimen of probiotic supplementation did not yield any clinically significant changes in BMI or laboratory parameters, including lipid and glucose profiles, when compared to the placebo [107]. These inconsistent findings underscore the imperative for further rigorously designed clinical trials to accurately assess the efficacy of probiotics in the treatment of MASH.

### 5.3. Comparative Analysis of Probiotic Strains in MASH Treatment

The comparative analysis of probiotic strains in the treatment of MASH represents a burgeoning field of research (Table 3). A systematic review and network meta-analysis have examined the impact of both traditional probiotics and next-generation probiotics (NGPs) on MASLD and MASH. The findings indicate that traditional probiotics primarily mitigate liver fat accumulation and inflammation by enhancing gut microbiota composition, strengthening intestinal barrier function, and modulating immune responses. Conversely, NGPs exhibit a more pronounced therapeutic potential, largely due to their direct effects on inhibiting oxidative stress and their capacity to increase the production of SCFAs [108]. Comparative studies of various probiotic strains in MASH management have elucidated their distinct effects. For instance, a study assessing the therapeutic efficacy of the genetically engineered probiotic Zbiotics (ZB183) in a MASH mouse model demonstrated that oral administration of Zbiotics for four weeks led to the downregulation of the cGAS-STING-related network, alleviated hepatic inflammation and steatosis, and enhanced colon health [101].

In a randomized controlled trial involving patients with MASH, a six-month supplementation with a probiotic containing *Lactobacillus acidophilus* and *Bifidobacterium lactis* resulted in a reduction in the APRI score. However, no significant differences were observed in liver fibrosis, steatosis, or inflammatory activity between the probiotic and placebo groups [103]. In a comparative study examining the effects of probiotics on cholesterol levels, liver morphology, and gut microbiota in obese mice, it was found that different probiotic strains and their combinations exhibited strain-dependent effects. Notably, a formulation comprising *B. animalis* VKL, *B. animalis* VKB, and *L. casei* IMV B-7280 effectively restored the liver morphological structure in obese mice, with various strains exerting differential effects on serum lipid profiles and gut microbiota composition [109].

Additionally, some studies have compared the efficacy of single-strain versus multi-strain probiotics. A meta-analysis of RCTs on the prevention of necrotizing enterocolitis (NEC) in preterm infants demonstrated that multi-strain probiotic formulations were more effective than single-strain probiotics in preventing severe NEC [110]. In a study examining the prophylactic impact of probiotics on oral mucositis, it was found that multi-strain probiotic formulations were significantly more effective than single-strain probiotics in mitigating severe oral mucositis [110].

Similarly, in the context of preventing NEC in preterm very low birth weight (PVLBW) infants, the use of multiple probiotic strains was associated with a substantial reduction in NEC incidence, evidenced by a pooled OR of 0.36. In contrast, single-strain probiotics utilizing *Lactobacillus* species demonstrated only a marginal effect in reducing NEC (OR of 0.60), without impacting mortality rates [111]. These findings imply that different probiotic strains may exhibit varying levels of efficacy in the management of MASH, with multi-strain probiotics potentially offering distinct advantages.

**Table 3:** Probiotics shown to ameliorate MASH in studies.

Probiotic Strains	Treatment Results	References
*L. fermentum*	Caspase 3 ↓, Caspase 9 ↓, STAT3 ↓, TNF-alpha ↓, apoptosis↓, IL-10 ↑, IL-6 ↓	[112]
*Bifidobacterium*	NF-κB ↓, pyroptotic-related genes ↓, LPS ↓, NLRP3 ↓, caspase-1 ↓, pro-IL-1β ↓, IL-1β ↓, GSDMD ↓	[113]
*Lactobacillus plantarum*	Hepatic inflammation ↓, apoptosis ↓, PNPLA3 ↓, SREBP-1c ↓, Gram-negative species ↓, bacterial translocation ↓	[114]
Zbiotics (ZB183)	cGAS-STING-related network ↓, hepatic TNF-α ↓, crypt length ↑, inflammatory cell infiltration ↓, colonic mucosa occluding ↑	[101]
Binary *Bacillus subtilis*	Liver inflammation ↓, IL-6 ↓, TNF-α ↓, IL-17 ↓, occluding ↑	[115]
*Akkermansia muciniphila*	Hepatic M1, γδT cells and γδT17 cells ↓, TLR2 ↓	[116]
Compound probiotics (*Lactobacillus plantarum* B7, *Lactobacillus rhamnosus* L34 and *Lactobacillus* *paracasei* B13)	Fat accumulation ↓, hepatocyte ballooning ↓, lobular inflammation ↓, CD14 ↓, TLR4 ↓, *Lactobacillus* ↑, hepatic fat droplets ↓, hepatic FFA levels ↓	[89]
*L. reuri*	Lipid profile ↓, oxidative stress ↓, inflammation ↓, Firmicutes and Bacteroidetes ↑	[117]
Compound probiotics VSL#3	Hepatic lymphocyte infiltration ↓, hepatic fat content ↓, insulin sensitivity ↑, Bacteroidaceae ↓, Porphyromonadaceae ↓, Helicobacteraceae ↓, Lachnospiraceae ↑	[118]
Compound probiotics (received microbiota from donors)	Steatosis ↓, glycemic ↓	[119]
Compound probiotics (*Bifidobacterium lactis*, *Lactobacillus bulgaricus* and *Streptococcus* *thermophilus*)	Lipid droplets ↓, SOD ↑, HDL-C ↓, LDL-C ↓, AST ↓, ALT ↓, TG ↓	[120]

SOD: superoxide dismutase; HDL-C: high-density lipoprotein cholesterol; AST: aspartate aminotransferase; ALT: alanine aminotransferase; TG: triglycerides; LDL-C: low-density lipoprotein cholesterol. ↑, upregulate; ↓, downregulate.

### 5.4. Synergistic Potential of Combination Therapies

Combination therapies incorporating probiotics have demonstrated potential in the treatment of MASH. A study investigated various synbiotic combinations that included both prebiotics and probiotics to address MASLD. Notably, a combination of fructooligosaccharides and probiotics (FOS + Pro) provided enhanced protection against liver degeneration induced by a Western diet. This synbiotic formulation resulted in the lowest increases in body weight, liver weight, and liver-to-body weight ratios. Furthermore, it significantly improved liver histopathological markers, decreased serum AST and cholesterol levels, and mitigated hepatic inflammation and steatosis [121].

In research focusing on primary liver cancer, both live suspensions and sonicated extracts of *Lactiplantibacillus plantarum* strains, used either as monotherapy or in conjunction with standard chemotherapeutics (sorafenib for HCC and gemcitabine for cholangiocarcinoma (CCA)), exhibited inhibitory effects on CCA and HCC cells. This combination therapy enabled a reduction in the required doses of chemotherapeutic agents, demonstrating pronounced synergistic effects [122].

A separate study explored the effects of combining a CCR2/CCR5 antagonist with an FGF21 analog in a rodent model of MASH. The combined treatment exhibited characteristics of both compounds during both short- and long-term administration, thereby enhancing beneficial outcomes across all parameters of steatohepatitis and fibrosis. Inhibition of CCR2/5 led to a reduction in circulating Ly6C+ monocytes and hepatic monocyte-derived macrophages, and decreased hepatic inflammation and fibrosis. Concurrently, FGF21 agonism resulted in reduced body weight, liver triglycerides, and histological MASH activity [123].

In a rat model of MASH, administration of both single-strain (*Lactobacillus plantarum* B7) and mixed-strain (*Lactobacillus rhamnosus* L34 + *Lactobacillus paracasei* B13) probiotics led to reductions in hepatic fat accumulation and inflammation, along with alterations in the gut microbiome [89]. Liver histology revealed a greater degree of fat accumulation, hepatocyte ballooning, and lobular inflammation in the MASH group, which showed improvement in the groups treated with probiotics [89].

Probiotics may have synergistic effects when used in conjunction with other therapeutic interventions. For instance, a systematic review and meta-analysis of 15 RCTs investigated the efficacy of pioglitazone in the management of MASH. The findings indicated that pioglitazone was effective in reducing fasting plasma glucose, ALT, AST, GGT, triglycerides, the HOMA-IR, and glycated hemoglobin A1c levels [124]. The potential for probiotics to enhance the therapeutic outcomes of pioglitazone or other pharmacological agents warrants further investigation to elucidate the benefits of such combinations.

## 6. Controversies, Challenges, and Future Directions

### 6.1. Debate on the Efficacy of Probiotics in Reversing MASH

The effectiveness of probiotics in the treatment of MASH remains a subject of ongoing debate. Certain studies have demonstrated beneficial outcomes; for instance, a pilot trial revealed that a probiotic cocktail comprising *Lactobacilli*, *Bifidobacteria*, and *Streptococcus thermophilus* resulted in a significant reduction (exceeding 20%) in serum ALT levels compared to the control group, suggesting a reduction in inflammation among MASH patients [125]. Furthermore, a proof-of-concept study indicated that probiotic treatment may decrease liver fat and AST levels in MASH patients. The IHTG content was significantly reduced in the probiotic group compared to the usual care group, with six subjects in the probiotic group experiencing a reduction in IHTG content by more than 30% from baseline, compared to two subjects in the usual care group [104]. In addition, in a mouse model of MASH induced by a high-fructose, high-fat diet, the administration of *Faecalibacterium prausnitzii* was found to enhance glucose homeostasis, prevent hepatic lipid accumulation, mitigate liver damage and fibrosis, and alleviate hepatic steatosis and inflammation [88].

Nevertheless, other research has yielded less definitive results. In a double-blind, placebo-controlled clinical trial involving 48 patients with MASH, supplementation with probiotics (*Lactobacillus acidophilus* and *Bifidobacterium lactis*) over a six-month period did not result in significant improvements in liver fibrosis, steatosis, or inflammatory activity, although a reduction in the APRI score was observed over time in the probiotic group [103]. Similarly, another double-blind, placebo-controlled study with 46 MASH patients demonstrated that 24 weeks of probiotic supplementation did not lead to any clinically significant changes in BMI or laboratory parameters, including lipid and glucose profiles. Notably, the number of patients with high cardiovascular risk (CVR), as determined by atherogenic indexes, decreased from baseline in both the probiotic and placebo groups, as did levels of PAI-1 and miR-122, with no significant differences observed between the two groups [107].

A randomized clinical trial involving obese children with MASH or MASLD indicated that probiotics did not offer any advantage over lifestyle modifications in ameliorating obesity-associated metabolic disturbances [126]. Although the probiotic group exhibited a statistically significant reduction in BMI from baseline, this reduction was not significant when compared to the placebo group. Notably, the placebo group experienced significant reductions in triglycerides, AST, ALT, the AST/ALT ratio, and alkaline phosphatase levels over the treatment period. Furthermore, transient elastography conducted on a subsample did not reveal significant improvements in either group, reinforcing the notion that probiotics may not provide additional benefits beyond lifestyle modifications for addressing obesity-related metabolic derangements in children [126].

A meta-analysis of RCTs investigating the impact of probiotics on MASLD revealed that probiotics exerted a beneficial regulatory effect on liver function, steatosis, blood glucose levels, insulin levels, and blood lipid levels. However, they did not produce significant improvements in BMI, inflammatory markers, or the homeostasis model assessment of insulin resistance [127].

Overall, probiotic efficacy in MASH is modulated by a triad of interdependent determinants: strain specificity, host heterogeneity (including metabolic comorbidities, gut dysbiosis, and genetic variants), and methodological limitations (such as short duration, small sample sizes, and surrogate endpoints). These factors collectively drive heterogeneous clinical outcomes through strain–host–disease interactions. These variable outcomes may be attributable to heterogeneity in study design, patient populations, probiotic strains, and treatment durations. Consequently, further well-designed, large-scale clinical trials are necessary to elucidate the true efficacy of probiotics in the context of MASH.

### 6.2. Regulatory and Safety Concerns in Probiotic Use

Regulatory and safety considerations are critical in the application of probiotics for MASH. While probiotics are generally regarded as safe, theoretical risks have been identified, including the potential for systemic infections, harmful metabolic activities, excessive immune responses in vulnerable individuals, gene transfer, and gastrointestinal side effects [128]. Whole-genome analyses of probiotic product isolates have identified the presence of genes associated with antimicrobial resistance, virulence factors, and toxic metabolites in certain strains, which may pose health risks [129]. Therefore, adherence to stringent manufacturing practices and the implementation of whole-genome sequencing to assess virulence, toxin, and antibiotic resistance genes are crucial to ensuring the safety of probiotic products [130].

The safety profile of probiotics is intricately linked to their intended application, the consumer’s potential susceptibility, dosage, duration of consumption, and method of administration [131]. For instance, in preterm infants, concerns arise regarding probiotic use due to their underdeveloped immune systems, although extensive trials have documented minimal adverse events [132]. A study investigating probiotic use in individuals with irritable bowel syndrome revealed that while probiotics were associated with a reduced incidence of persistent symptoms, there was a higher incidence of adverse events among those treated with probiotics [133]. It is crucial to assess probiotic safety in certain patient groups and conduct more research on their long-term effects [134].

The regulatory landscape for probiotics is inconsistent across different countries, with no global consensus on the criteria for establishing their safety for use in food and supplements [135]. In certain jurisdictions, probiotics are categorized as food supplements, with regulatory emphasis placed on the legitimacy of claims rather than on efficacy, safety, and quality. This lack of rigorous regulation can result in challenges such as inaccurate labeling, exaggerated claims, and the risk of pathogenic contamination in probiotic products [136]. Furthermore, there is a deficiency in the standardization of safety reporting in clinical studies, which complicates the establishment of a robust evidence base regarding probiotic safety.

### 6.3. Barriers to Patient Adherence and Acceptance

Patient adherence and acceptance are critical considerations in the application of probiotic therapy for MASH. A study investigating the use of probiotics as an adjunct to sequential *Helicobacter pylori* eradication therapy observed reduced rates of first-week treatment non-compliance due to diarrhea in the probiotic group compared to the non-probiotic group, suggesting that probiotics may enhance treatment compliance in certain instances [137].

Conversely, in research involving pediatric patients with ulcerative colitis, while the majority expressed willingness to pursue fecal microbiota transplant (FMT) due to its perceived “natural” qualities and the ineffectiveness of conventional medications, pre-treatment apprehensions regarding physical discomfort associated with FMT administration were prevalent [138]. Similarly, a study on patients with MASLD identified factors such as difficulty in remembering to take probiotics, side effects, and cost as potential barriers to patient adherence [139]. Addressing these factors is essential for the effective implementation of probiotic therapy in MASH patients.

### 6.4. Future Innovations and Directions

Emerging probiotic formulations are currently under investigation for the treatment of MASH. The field is investigating engineered probiotics via synthetic biology to target specific metabolic pathways and produce beneficial metabolites. These probiotics could secrete anti-inflammatory cytokines or enzymes to modulate gut microbiota and improve liver function in MASH. Genetically engineered probiotics like ZB183 have shown potential benefits; in a MASH murine model, Zbiotics downregulated the cGAS-STING pathway, reducing liver inflammation and steatosis and improving colonic health, indicating its potential as a novel therapeutic candidate [101]. Furthermore, NGPs like *Faecalibacterium prausnitzii* show considerable promise. In one study, four strains of *F. prausnitzii*, isolated from healthy individuals, were administered to mice with MASH induced by a high-fructose, high-fat diet. The findings revealed that *F. prausnitzii* supplementation enhanced glucose homeostasis, inhibited hepatic lipid accumulation, and mitigated liver inflammation [88].

Emerging probiotic technologies present novel opportunities for the modulation of the gut microbiome in the context of MASH. Nanoencapsulation of probiotics has been identified as a promising approach to enhance the stability and bioavailability of these microorganisms [140]. For instance, dual-targeted nanoparticles utilizing *Lactobacillus rhamnosus* bacterial ghosts have been developed for the delivery of astaxanthin as an intervention for MASH [141]. In vitro studies have demonstrated that these nanoparticles significantly mitigate LPS-induced reactive oxygen species (ROS) production and apoptosis. Concurrently, in vivo studies have shown improved intestinal accumulation and efficacy in alleviating MASH symptoms, including reductions in triglycerides, free fatty acids, and malondialdehyde levels [141].

Moreover, the combination of probiotics with prebiotics (synbiotics) or postbiotics may provide augmented therapeutic effects. For instance, a synbiotic formulation could be designed to selectively utilize the co-administered microorganisms, potentially leading to more effective modulation of the gut microbiota and improved clinical outcomes in patients with MASH.

Future research on probiotics for MASH could be directed towards several promising areas. A significant challenge in the application of probiotics for MASH lies in their formulation and delivery. Probiotics must withstand the adverse conditions of the gastrointestinal tract, such as the low pH of the stomach and exposure to bile salts in the small intestine, to effectively reach the gut and exert their beneficial effects [142]. One innovative approach is the encapsulation of probiotics using liposome-mediated techniques. This method offers protection to probiotics against environmental factors, gastric acid, and bile, thereby enhancing their viability, release, and absorption within the intestines. Nevertheless, further investigation is required to optimize the encapsulation process, elucidate the impact of the digestive process on the efficacy of liposomes and probiotics, and address challenges related to large-scale production and cost-effectiveness [143].

To address this, encapsulation techniques employing biopolymers like cellulose, chitosan, and alginate have been developed to safeguard probiotics during gastrointestinal transit [142]. Nonetheless, these techniques encounter obstacles, including maintaining probiotic viability during the dehydration process and ensuring targeted release at the appropriate site within the gut [144]. Furthermore, the choice of formulation can significantly impact the stability and functionality of probiotics. For instance, while spray-dried probiotic microcapsules have demonstrated potential, the harsh environmental conditions encountered during drying and digestion can markedly reduce cell viability, underscoring the necessity for further optimization of formulation and delivery methods [145]. Another promising avenue of research involves elucidating how probiotics influence the production and composition of EVs, as well as the autophagy pathway, which may unveil new targets for the treatment of MASH [146].

There is an urgent need for well-designed clinical trials to determine the effectiveness of probiotics in managing MASH. These studies should assess different probiotic strains, dosages, treatment durations, and patient demographics to create clear clinical guidelines. Understanding how probiotics interact with the gut microbiome, immune responses, and liver cells at the molecular level is crucial for developing effective treatments. Research should also consider the long-term effects of probiotics, including potential side effects and resistance. Additionally, combining probiotics with other therapies could improve treatment outcomes.

## 7. Conclusions

Based on the evidence synthesized in this review, probiotics exhibit potential to modulate the gut–liver axis by restoring microbial balance, enhancing intestinal barrier integrity, suppressing pro-inflammatory signaling pathways, and ameliorating metabolic dysregulation in MASH. Despite the limitations of current pharmacotherapy, probiotic interventions present a multimodal therapeutic strategy that targets interconnected pathogenic mechanisms, including dysbiosis-induced endotoxemia, bile acid disturbances, and lipotoxicity. This approach is congruent with the complex pathophysiology of MASH, leveraging the systemic influence of the gut microbiome on metabolic and inflammatory processes. Nevertheless, clinical outcomes demonstrate heterogeneity due to variations in probiotic formulations, treatment protocols, and patient-specific factors, underscoring unresolved controversies regarding efficacy and safety profiles. Future advancements ought to prioritize the implementation of rigorously designed clinical trials to establish standardized protocols, in conjunction with the development of innovative delivery systems and personalized microbiota-guided approaches. These developments are crucial for validating long-term therapeutic benefits and integrating probiotics into comprehensive management strategies for MASH.

## Data Availability

No new data were created or analyzed in this study. Data sharing is not applicable to this article.
